# Development of g-C_3_N_4_–TiO_2_ heterojunction for photocatalytic degradation of rhodamine B dye and production of hydrogen

**DOI:** 10.1039/d6ra03962f

**Published:** 2026-08-28

**Authors:** Asif Jamil, Marcus Vinicius Castegnaro, Muhammad Saeed, Muhammad Rameez Khan Khattak, Anderson Thesing, Larissa Stein Godoi, Giovanna Machado, Sherdil Khan

**Affiliations:** a Institute of Physics, Universidade Federal do Rio Grande do Sul Av. Bento Gonçalves 9500, Bairro Agronomia Porto Alegre RS 91501-970 Brazil marcus.castegnaro@ufrgs.br sherdil.khan@ufrgs.br; b Department of Chemistry, Government College University Faisalabad Faisalabad Pakistan; c Laboratory of Microscope and Microanalysis, Northeast Center for Strategic Technologies (CENETE) Avenida Prof. Luís Freire, 1, Cidade Universitária Recife Pernambuco 50740-540 Brazil

## Abstract

Organic dyes are released into wastewater from various industries and pose a significant risk to living organisms. The eradication of organic dyes from aqueous systems by photocatalysis has received the attention of researchers promoting environmental sustainability. In this study, g-C_3_N_4_, TiO_2_, and their composites in a 1 : 1, 1 : 2, and 2 : 1 ratio were prepared and characterized. The prepared composites were tested for the catalytic elimination of aqueous rhodamine B dye and for the production of hydrogen using an aqueous solution of triethanolamine (TEOA) under visible light. It was noticed that ∼65, 22, 87, 100, and 78% of the rhodamine B dye (50 ppm, 35 mL) degraded after 2 h reaction with 35 mg g-C_3_N_4_, TiO_2,_ and their composites in a 1 : 1, 1 : 2, and 2 : 1 ratio, respectively. The visible light-assisted production of hydrogen from water vapor splitting was 110, 32, 2597, 3463, and 1791 µmol g^−1^ with g-C_3_N_4_, TiO_2_, and their composites in a 1 : 1, 1 : 2, and 2 : 1 ratio, respectively. The integration of g-C_3_N_4_ with TiO_2_ significantly boosted the catalytic role due to the synergistic interaction between the individual constituents. The prepared composite exhibited similar photocatalytic activity in three consecutive cycles of rhodamine B degradation and four consecutive cycles of hydrogen production, indicating the highly stable nature of the fabricated composite. It was concluded that g-C_3_N_4_–TiO_2_ can be used as an effective catalyst in remediation of wastewater contaminated with organic pollutants and the production of hydrogen in visible light.

## Abstract 2

The synergistic effect of type-II heterojunctions between g-C_3_N_4_ and TiO_2_ enhances charge separation, thereby improving hydrogen production and organic dye degradation.

## Graphical abstract

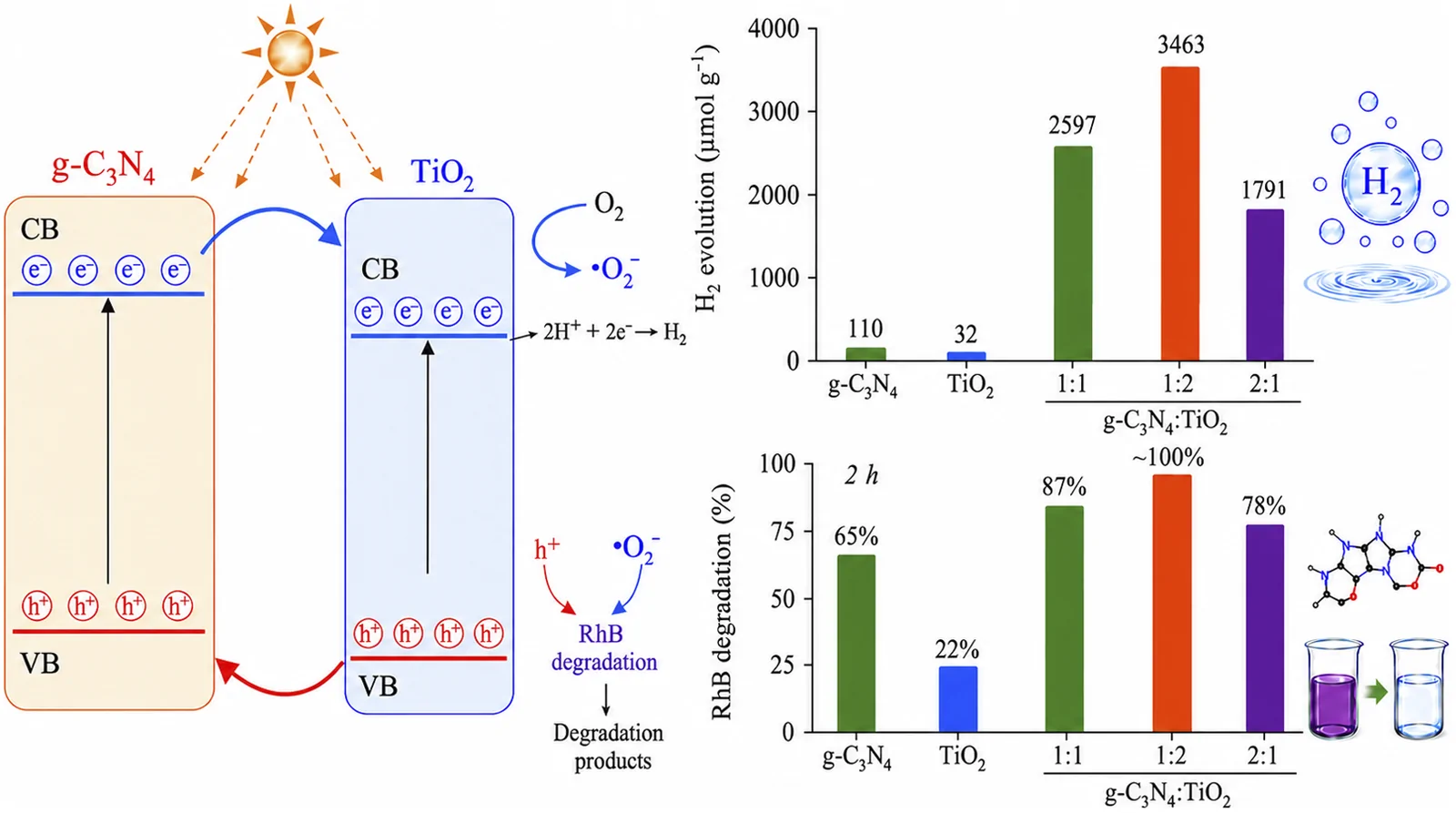

## 1 Introduction

The rapid increase in urbanization and industrialization has created many problems, such as environmental contamination, energy crises, generation of engineering waste mud, overcrowding, resource depletion, *etc.* The first two problems related to the environment and energy are problems of significant concern around the world.^1^ A huge amount of wastewater is released from the industries around the globe. This industrial wastewater contains numerous toxic pollutants, including organic and inorganic compounds.^2–5^ Organic dyes, as aqueous pollutants, have been a major concern around the world because these pollutants impose a substantial impact on living organisms and the ecosystem. Due to stability and toxic effects such as teratogenic, genotoxic, and carcinogenic effects, organic dyes have been considered as persistent organic pollutants. A wide range of dyes is extensively used in different industries such as printing, textile coloration, pharmaceuticals, cosmetics, foods, *etc.* The production of dyes has been reported as 175 000 tons per year around the world, and 31% of these dyes are discharged into water systems.^6^ Hence, these industries release a substantial amount of dyes in their wastewater effluents.^7,8^ Therefore, the removal of these toxic dyes from the aqueous system is crucial. Traditional methods often fail in the removal of dye pollutants from aqueous systems. Consequently, there is a need to develop an effective, sustainable, and advanced methodology for the eradication of dyes from the environment. Photocatalysis, which employs light to activate a semiconductor metal oxide-based catalyst, is an environmentally friendly and promising technique that breaks down the organic pollutants into simple molecules. A wide range of semiconductor metal oxides have been utilized as catalysts in photocatalysis. Titanium dioxide (TiO_2_) is one of them that has sparked a significant interest due to its extraordinary properties, such as low production cost, strong oxidizing power, high stability, and non-toxicity.^9–12^ However, TiO_2_, being a wide band gap substance, is not a successful photocatalyst under sunlight or visible light irradiation. Solar energy is one of the most abundant renewable resources.^13^ Therefore, it is crucial to develop a photocatalyst that can perform under visible light irradiation. Different strategies have been adopted to improve the activity of TiO_2_. The construction of a heterojunction by combining it with another narrow-band gap semiconductor is an effective protocol. The g-C_3_N_4_ (graphitic carbon nitride) received considerable focus from researchers in the construction of a heterojunction with TiO_2_ due to well-matched band levels of both substances. It is a narrow band gap substance having suitable characteristics such as low cost, non-toxicity, good abrasion resistance, low density, and good stability. Its band gap is ∼2.7 eV.^14^ The construction of a heterojunction by its integration with TiO_2_ not only improves harvesting of visible light but also improves the migration of positive holes and electrons through their transfer between them. Both of these characteristics boost the effectiveness of g-C_3_N_4_–TiO_2_.^9^ Furthermore, visible-light-responsive g-C_3_N_4_–TiO_2_ heterojunctions have also attracted considerable attention for solar-driven hydrogen production. Hydrogen is regarded as one of the most attractive next-generation energy carriers due to its ability to support sustainable energy systems and alleviate environmental challenges associated with conventional fuels. Among the various hydrogen production routes, photocatalytic water splitting is particularly attractive because it directly converts abundant solar energy into chemical fuel under mild operating conditions. Therefore, the development of highly active photocatalysts responsive to visible light are essential for advancing sustainable hydrogen production and overcoming pressing energy and environmental issues.^15,16^

The aims of this study are to (i) enhance the performance of TiO_2_ catalyst through coupling with g-C_3_N_4_, (ii) optimize the composition of the g-C_3_N_4_–TiO_2_ heterostructure, and (iii) design a high-performance photocatalyst capable of simultaneously facilitating dye decomposition and hydrogen generation. Beyond performance evaluation, this work brings a simultaneous investigation of photocatalytic hydrogen evolution and pollutant degradation, combined with photoelectrochemical characterization to elucidate charge-transfer dynamics at the semiconductor interface. Furthermore, band alignment analysis was performed to verify the construction of a Type-II heterojunction, providing mechanistic insight into the improved performance of the optimized g-C_3_N_4_–TiO_2_ system.

## 2 Experimentation

### 2.1 Synthesis of photocatalyst

g-C_3_N_4_ was prepared *via* high-temperature polycondensation of melamine. About 10 g of melamine powder was subjected to high-temperature treatment for 120 min at 550 °C at the rate of 3 °C min^−1^. g-C_3_N_4_ was obtained in the form of yellow powder after cooling naturally to room temperature.

TiO_2_ was prepared *via* the sol–gel method. The precursor was tetrabutyl titanate. 40 mL of anhydrous ethanol and 17 mL of tetrabutyl titanate were mixed dropwise under continuous stirring for 30 min. In a separate 250 mL flask, 2.5 mL of distilled water and 60 mL of anhydrous ethanol were mixed, and the resultant solution was acidified to pH 1–2 with HCl. Both solutions were mixed slowly while stirring continuously. The stirring of the resulting mixture continued for another 2 h. The reaction was stabilized by keeping the obtained sol at room temperature for 24 h. Afterwards, the substrate was dried at 80 °C to get the TiO_2_ precursor. The precursor was converted to TiO_2_ by 2 hours of calcination at 500 °C.

**Fig. 1:**
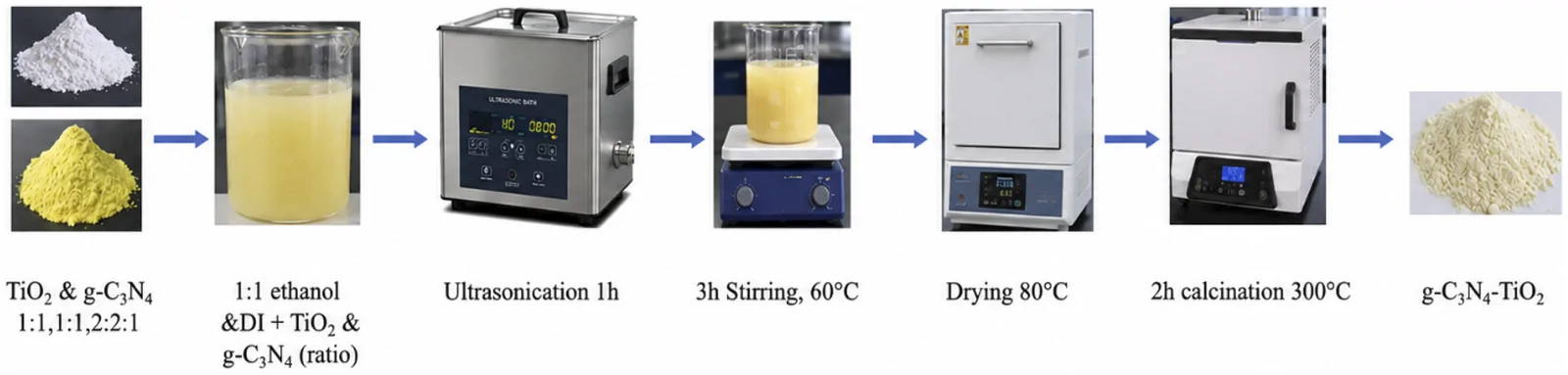
Schematic representation of the preparation route employed for the fabrication of the g-C_3_N_4_–TiO_2_ heterostructure.

To fabricate the heterostructure, the prepared g-C_3_N_4_ was mixed with TiO_2_ with mass ratios of 1 : 1, 1 : 2, and 2 : 1. The required amounts of both materials were suspended in 60 mL of a 1 : 1 water–ethanol mixture. The suspension was ultrasonicated for 60 min for uniform dispersion of the particles, followed by stirring at 60 °C for 180 min. Then, it was dried at 80 °C in an oven to remove the solvent, yielding a mixed g-C_3_N_4_–TiO_2_ powder. Finally, it was calcined at 300 °C for 120 min, followed by natural cooling to get g-C_3_N_4_–TiO_2_ composites (Fig. 1).

### 2.2 Preparation of photoelectrodes and PEC measurements

A 40 mg sample of the photocatalyst and 10 mg of iodine were dispersed in 40 mL of acetone, followed by ultrasonication for 1 h. Then, the suspension was magnetically stirred for 1 h to obtain a homogeneous mixture. The photoelectrode was fabricated by depositing the suspension onto the cleaned FTO substrate using electrophoretic deposition (EPD) at an applied voltage of 30 V for 2 min. The deposited film was then dried on a hot plate at 200 °C before photoelectrochemical (PEC) measurements.

### 2.3 Characterization of materials

The crystalline characteristics of the obtained materials were evaluated by XRD measurements carried out on a Rigaku Ultima IV instrument. The surface morphology was examined using a Zeiss EVO MA10 scanning electron microscope (SEM), and images were acquired using a secondary electron (SE2) detector at an accelerating voltage of 15 kV. The optical properties were investigated using a Cary 5000 UV-Vis spectrophotometer for the wavelength range of 200 to 800 nm by diffused reflectance and the bandgaps were estimated by Tauc plots. X-ray photoelectron spectroscopy (XPS) was used for determination of elemental composition and oxidation states of the constituent elements using a Scienta Omicron ESCA+ system. High-resolution spectra were collected with a pass energy of 20 eV and analyzed using CasaXPS software. Functional groups were identified by Fourier-transform infrared (FTIR) spectroscopy on a PerkinElmer Spectrum 1000 spectrometer. Photoluminescence (PL) spectra were recorded using a Cary Eclipse fluorescence spectrophotometer to evaluate the charge recombination behavior.

The PEC characterization was performed in a 0.1 M sodium sulfate Na_2_SO_4_ electrolyte using the prepared photoelectrode. Mott–Schottky measurements were performed at 1000 Hz in the potential range of −0.6 to 0.8 V *vs.* Ag/AgCl under light conditions (AM 1.5G filter, 100 mW cm^−2^). The donor density (*N*_D_) was estimated from the slope of the Mott–Schottky plots according to eqn (1):1
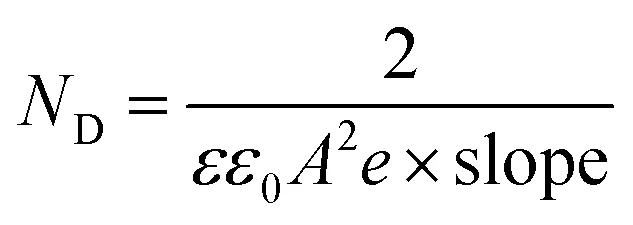
where *ε* is the dielectric constant of the semiconductor, *ε*_0_ is the vacuum permittivity, (*e*) is the elementary charge, and *A* corresponds to the geometric electrode. The conduction band edge potential *E*_CB_ was subsequently estimated from the flat-band potential *V*_fb_ using the calculated donor density values and the relationship between the Fermi level and the conduction band edge for n-type semiconductors. Open-circuit potential decay (OCPD) measurements were conducted to study the charge carrier dynamics. The charge carrier dynamics were evaluated by open-circuit potential decay (OCPD) measurements under 1 Sun illumination. The dark decay region of the OCPD curves was fitted using a biexponential function according to eqn (2):

2 *V*(*t*) = *V*_0_ + *A*_1_ e^(−*t*/*τ*_1_)^ + *A*_2_ e^(−*t*/*τ*_2_)^

where (*V*_0_) is the equilibrium potential, (*A*_1_) and (*A*_2_) are fitting constants, and *τ*_1_ and *τ*_2_ represent the characteristic decay constants associated with fast and slow charge transfer processes, respectively. The fitting parameters and their corresponding uncertainties are summarized in Table S1.

### 2.4 Photocatalysis

Rhodamine B (RhB) was employed as prob pollutant to investigate the degradation capability of the g-C_3_N_4_–TiO_2_ composites and their pristine constituents under visible light. A 50 ppm solution of RhB was used for this purpose. In a typical degradation process, 35 mL of dye solution was charged with 35 mg of catalyst, and then it was sonicated for 30 min for homogeneous dispersion. Before the photocatalytic reaction, the mixture was agitated in the dark for 60 min for getting the adsorption–desorption equilibrium. Then, the mixture was exposed to irradiation using a 100 W visible light lamp. The lamp was fitted to an AM 1.5G filter to maintain the solar condition. During the photocatalytic reaction, samples were collected every 20 minutes and centrifuged for 6 minutes for separation of catalyst particles. The reaction was monitored through measurement of the absorbance of RhB at 554 nm with a UV-M51 spectrophotometer.

For catalytic activity of g-C_3_N_4_–TiO_2_ composites and individual components in the production of hydrogen energy, 20 mg each was suspended in a mixture of 18 mL of deionized water and 2 mL of triethanolamine (TEOA) in separate experiments. The suspension was sonicated for 30 min for homogeneity. It was then placed in a 50 mL airtight quartz reactor. Argon gas was purged to remove dissolved oxygen. Photocatalytic H_2_ evolution was performed in visible light of 100 mW cm^−2^ intensity using a 300 W Xe lamp. It was fitted to an AM 1.5 G filter. The reaction continued for 4 h. The evolved hydrogen was measured using a TCD detector equipped in a Shimadzu GC-2014. The first measurement was taken in the dark, followed by measurements every hour during irradiation.

## 3 Results and discussion

### 3.1 Characterization

**Fig. 2:**
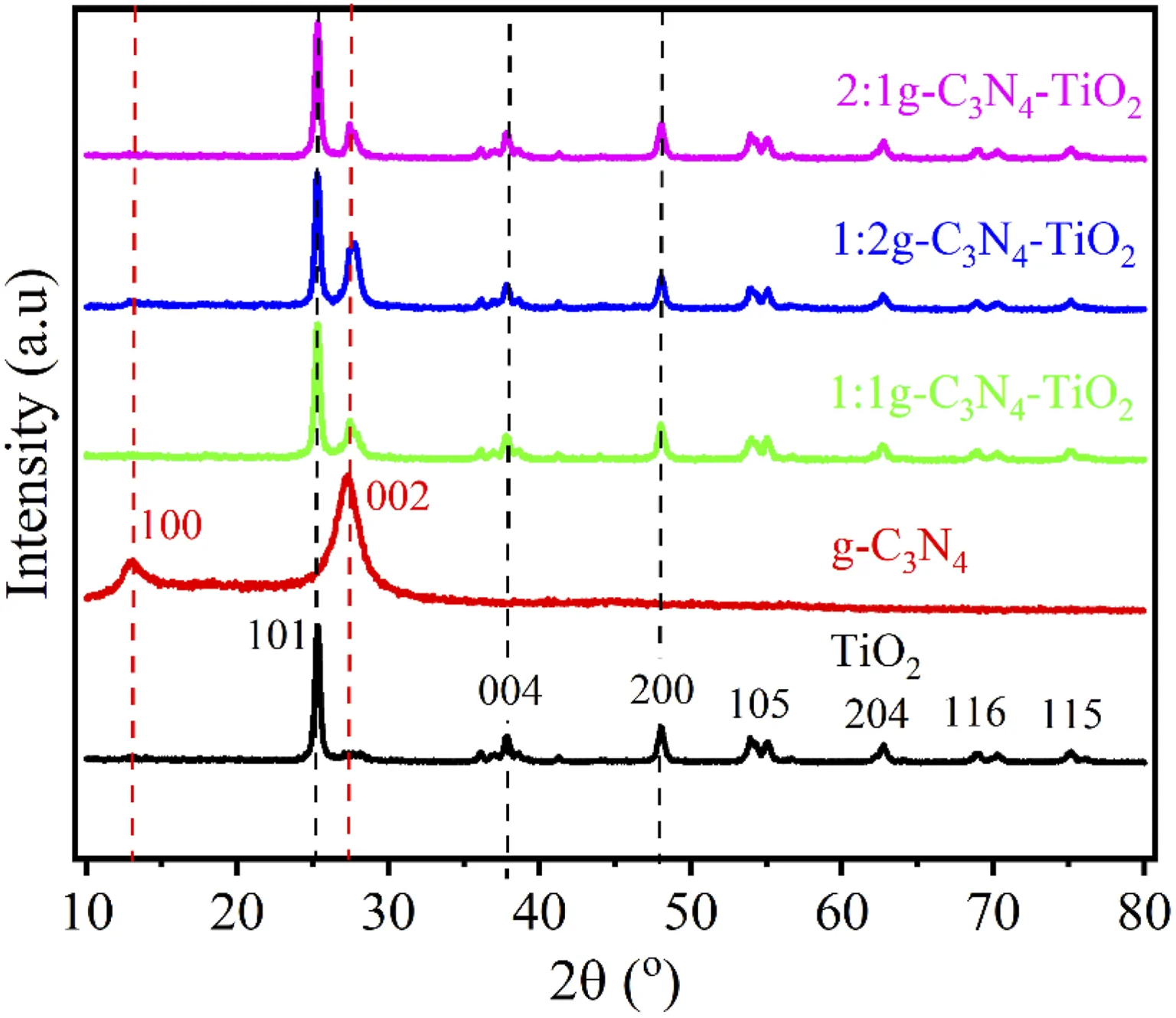
XRD spectra of TiO_2_, g-C_3_N_4_, and g-C_3_N_4_–TiO_2_ samples. The observed peaks match with peaks of g-C_3_N_4_ and TiO_2_, which confirms the successful fabrication of the heterojunction without the appearance of secondary crystalline phases, indicating effective integration of both components.

Fig. 2 depicts XRD spectra of various samples. The presence of the sharp peaks in XRD spectra at 2*θ* ∼13°, 25°, 27°, 38°, 48°, 54°, 55°, 63°, 71°, and 75° confirms that g-C_3_N_4_–TiO_2_ composites have a crystalline nature. According to JCPDS 87-1526, the peaks at 2*θ* ∼13° and 27° indicate the presence of g-C_3_N_4_ in composites. These peaks represent the (100) and (002) *hkl* planes.^17^ Similarly, the XRD peaks observed at 2*θ* ∼25°, 38°, 48°, 54°, 55°, 63°, 71°, and 75° represent the existence of TiO_2_ in the prepared samples (JCPDS 78-2486).^18^ These peaks represent the (101), (004), (200), (105), (211), (204), (116), and (215) *hkl* planes, respectively. The XRD analyses of g-C_3_N_4_–TiO_2_ composites showed that there is no appearance of a new phase. Hence, the prepared g-C_3_N_4_–TiO_2_ samples are hybrids of both components.^19,20^

**Fig. 3:**
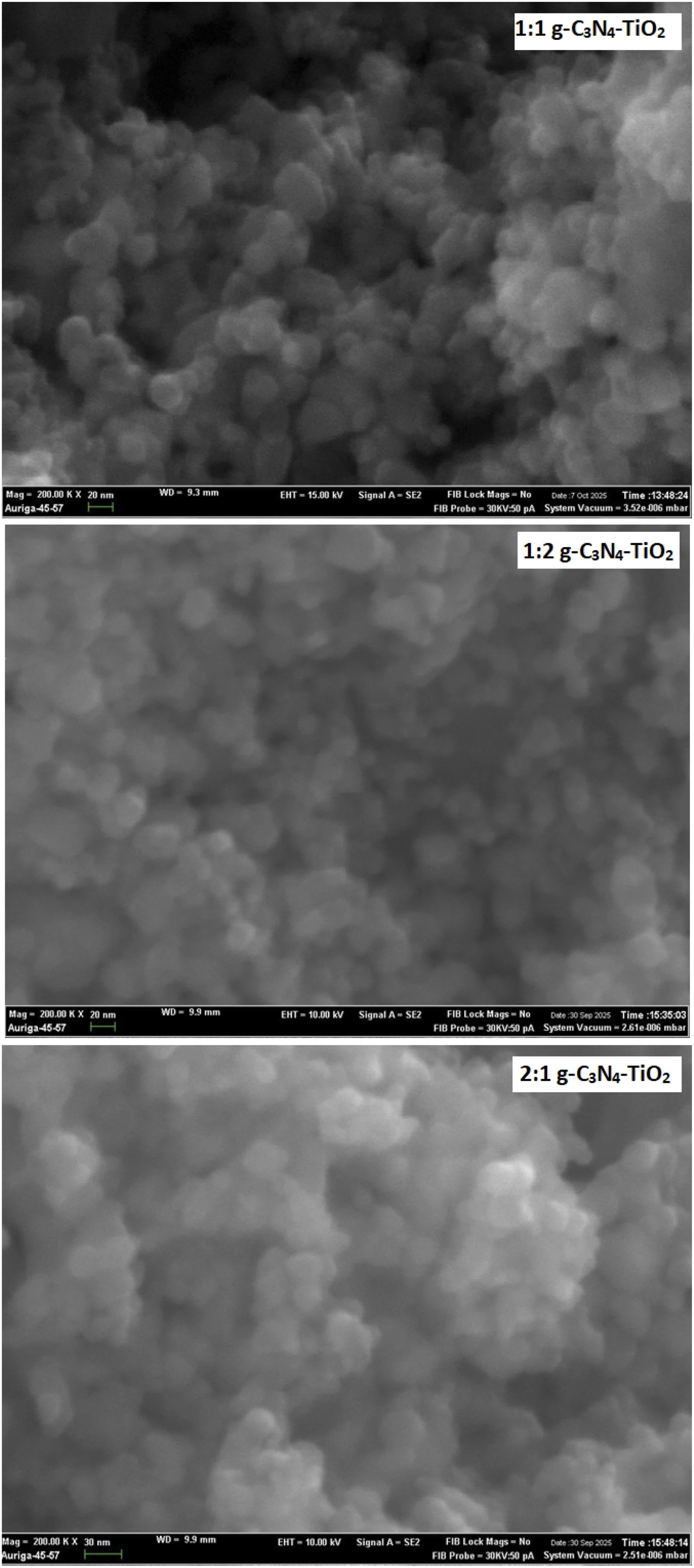
SEM analysis of g-C_3_N_4_–TiO_2_ composites. These images reveal that particles are predominantly spherical and uniformly distributed with slight agglomeration.

Fig. 3 shows the results obtained from a scanning electron microscope. It is evident from micrographs that the particles are predominantly spherical and uniform in shape. It can also be observed that the particles are slightly agglomerated. The uniform morphology of the particles is an indication of effective control of the nucleation and growth process during the synthesis of particles. This morphological stability is crucial for photocatalytic applications, as it ensures reproducibility and predictable performance.

UV-Vis diffuse reflectance spectroscopy (DRS) was performed to evaluate the optical properties of the samples. The observed UV-visible spectra are given in Fig. S1. It can be noted that the g-C_3_N_4_–TiO_2_ composites absorb in visible region. The occurrence of absorbance in visible region of the spectrum is due to the successful integration of g-C_3_N_4_ with TiO_2_.

**Fig. 4:**
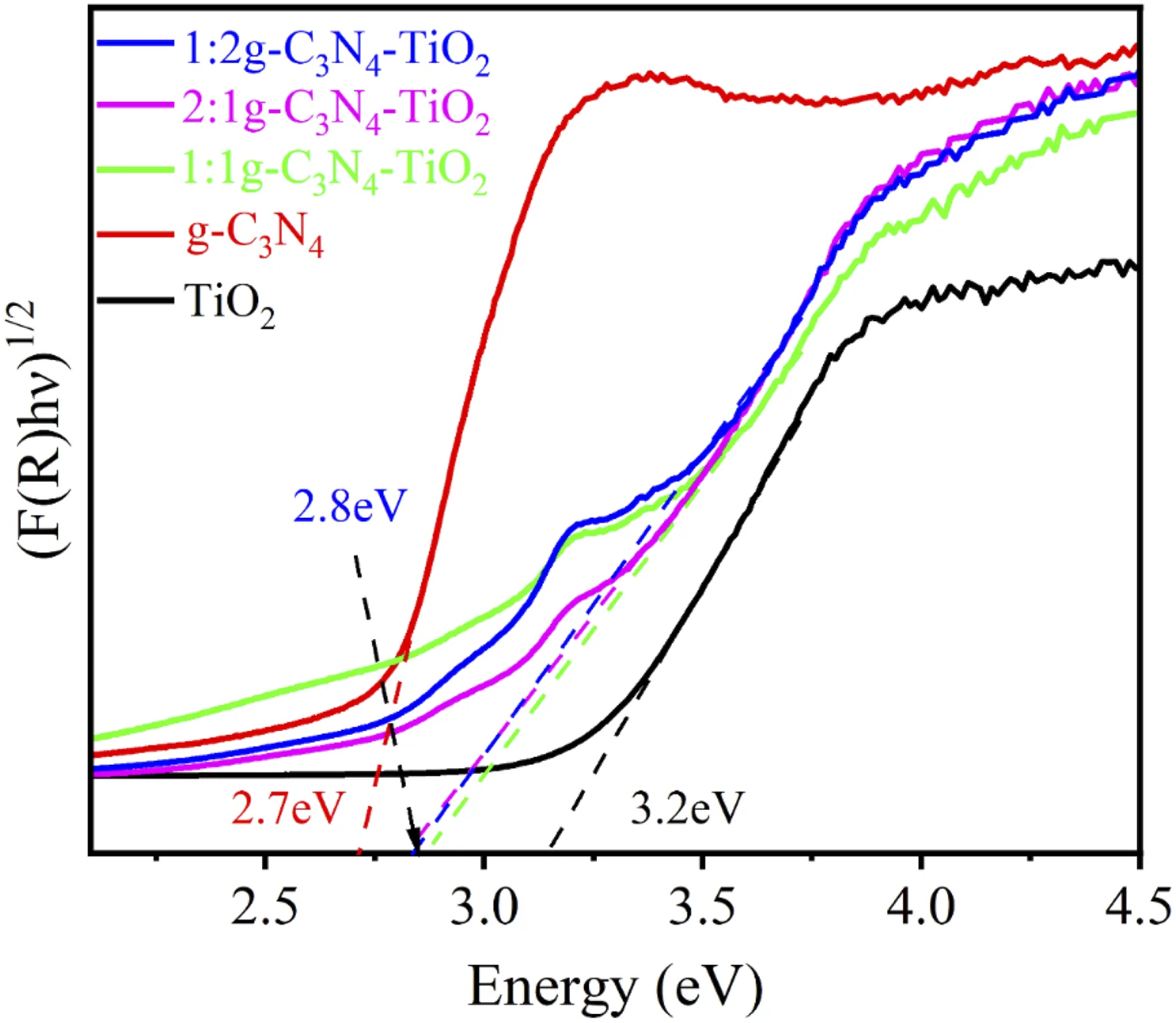
Tauc plots of g-C_3_N_4_–TiO_2_ composites and their individual components. The reduced band gaps of the heterojunction composites compared with pure TiO_2_ demonstrate enhanced visible-light responsiveness of the composites.

The integration of g-C_3_N_4_ with TiO_2_ extends the absorption to visible light region and boosts catalytic effectiveness. The optical band gap was calculated from the UV-Vis diffuse reflectance spectra using the Kubelka–Munk transformation and Tauc plot analysis, as shown in Fig. 4. The cut-off segment on the energy axis, obtained by extrapolating the linear sector of (*F*(*R*)*hν*)^1/2^*vs. hν*, yields the band gap of the sample.^21,22^ The value of the band gap of TiO_2_, g-C_3_N_4_, and their composites in 1 : 1, 1 : 2, and 2 : 1 ratios was calculated as 3.2, 2.7, 2.86, 2.83, and 284 eV, respectively. These band gap values of g-C_3_N_4_–TiO_2_ heterostructures are lower than the TiO_2_ band gap, which confirms that the combination of these components narrows the band gap and improves the utilization of visible light. The values of the band gap are reduced due to synergistic interaction between g-C_3_N_4_ and TiO_2_.

**Fig. 5:**
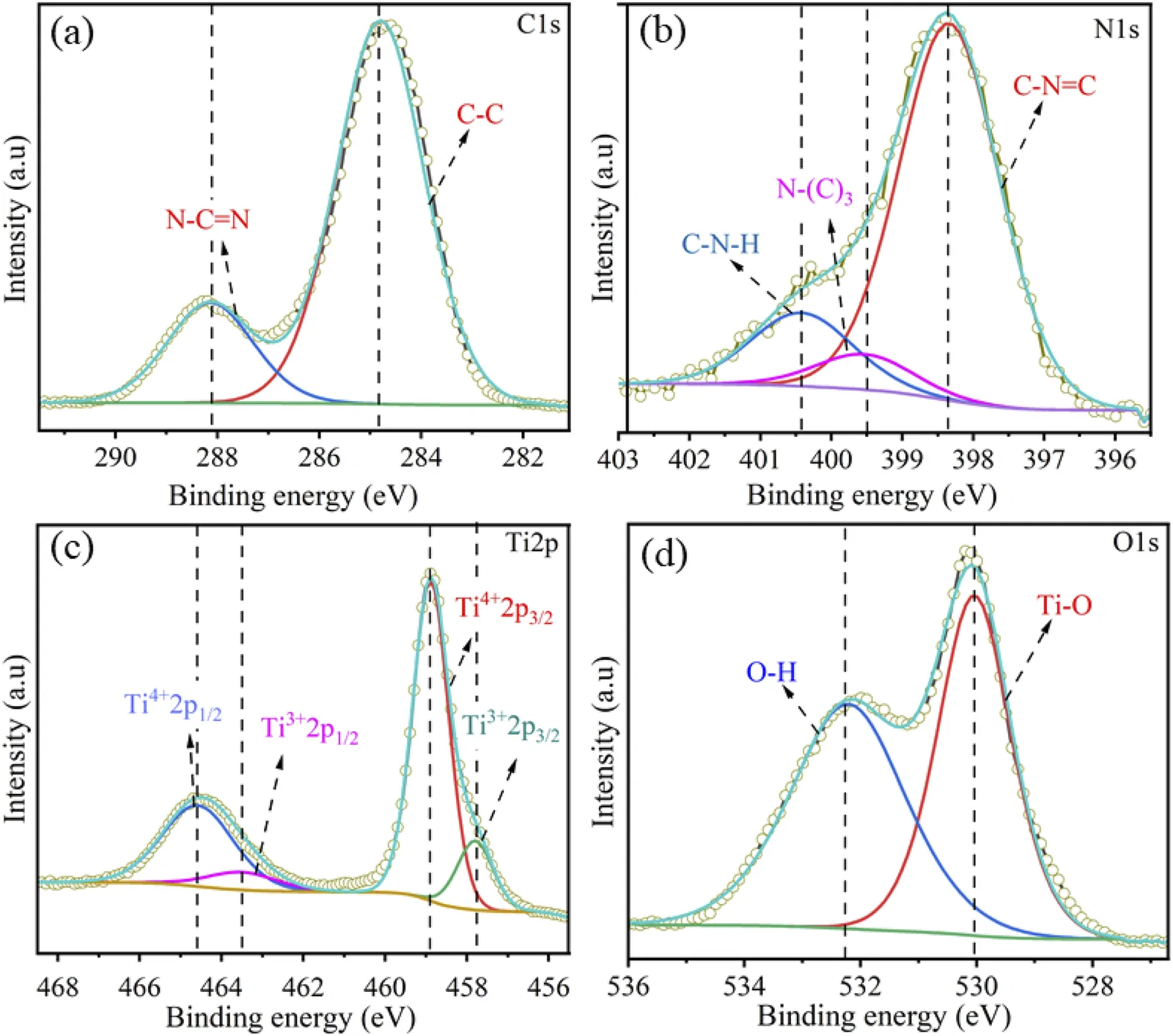
High-resolution XPS spectra of the 1 : 2 g-C_3_N_4_–TiO_2_ composite: (a) C 1s, (b) N 1s, (c) Ti 2p, and (d) O 1s.

Elemental composition and characteristics of surface and molecular interactions were investigated through XPS analysis. Fig. 5 displays the XPS spectra of the components of 1 : 2 g-C_3_N_4_–TiO_2_. The C 1s is deconvoluted into two peaks, Fig. 5(a), one at 284.8 eV and the other at 288.1 eV binding energy. The peak at 284.8 eV represents the adventitious carbon C–C bond^23,24^ and the peak at 288.1 eV is a characteristic of the sp^2^ hybridized N–C=N bonds in the g-C_3_N_4_ network.^25,26^

The N 1s peak is deconvoluted into three peaks: 398.4 eV, 399.5 eV, and 400.9 eV, Fig. 5(b). The peak at 398.4 eV is assigned to the sp^2^-hybridized nitrogen (C=N–C), the peak centered at 399.5 eV corresponds to tertiary nitrogen atoms bonded to three carbon atoms (N–(C)_3_), and the peak at 400.9 eV is attributed to amino groups (C–N–H).^26^ The high-resolution Ti 2p spectrum was fitted with four peaks corresponding to Ti^4+^ and Ti^3+^ species Fig. 5(c). The peaks at 458.9 and 464.6 eV are assigned to Ti^4+^ 2p_3/2_ and Ti^4+^ 2p_1/2_, confirming the presence of TiO_2_. The peaks at 457.8 and 463.5 eV are attributed to Ti^3+^ 2p_3/2_ and Ti^3+^ 2p_1/2_, indicating the presence of oxygen vacancies or surface defects. The O 1s XPS spectrum also exhibits two peaks, one at 530 eV and the other at 532.2 eV, Fig. 5(d). The first peak represents the Ti–O bond, while the second represents the O–H adsorbed on the sample. Hence, the XPS analysis confirms the successful integration of TiO_2_ and g-C_3_N_4_.^27–31^

**Fig. 6:**
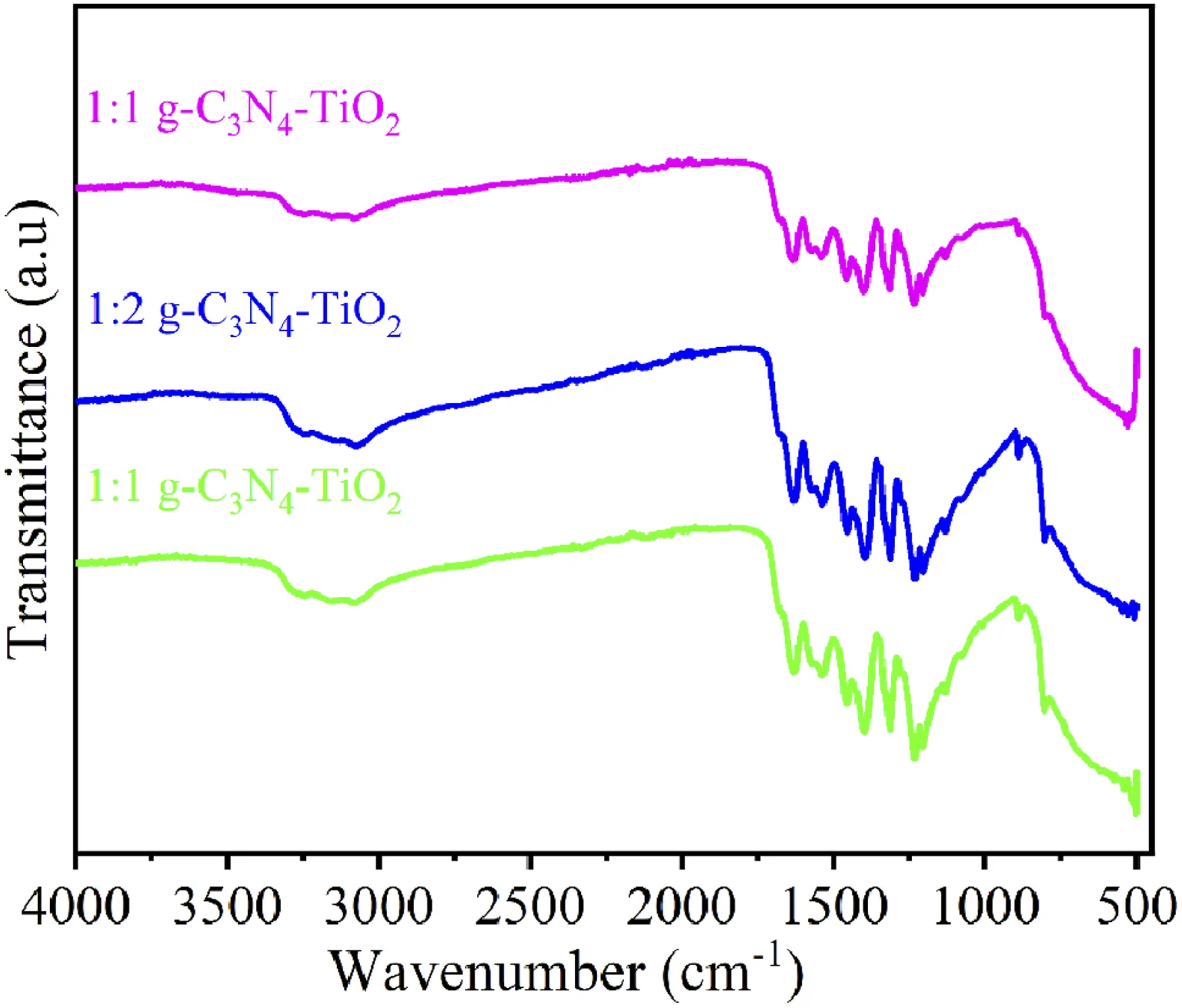
FTIR analysis of g-C_3_N_4_–TiO_2_ composites. Characteristic absorption bands confirm the coexistence of g-C_3_N_4_ and TiO_2_ in the composite structure.

The functional groups over g-C_3_N_4_–TiO_2_ were analyzed using FTIR spectroscopy. Fig. 6 shows the FTIR spectra. The absorption peaks over 500–700 cm^−1^ correspond to stretching modes of Ti–O and Ti–O–Ti. The signal at 808 cm^−1^ is related to the stretching of the heptazine arrangement. The occurrence of peaks over 1200–1450 cm^−1^ corresponds to sp^3^ C–N linkage. A signal detected at 1631 cm^−1^ indicates the stretching movement of sp^2^ C=N.^32,33^ The broad absorption peaks over 3350–3450 cm^−1^ are attributed to the O–H group.^34^

### 3.2 Photocatalysis

**Fig. 7:**
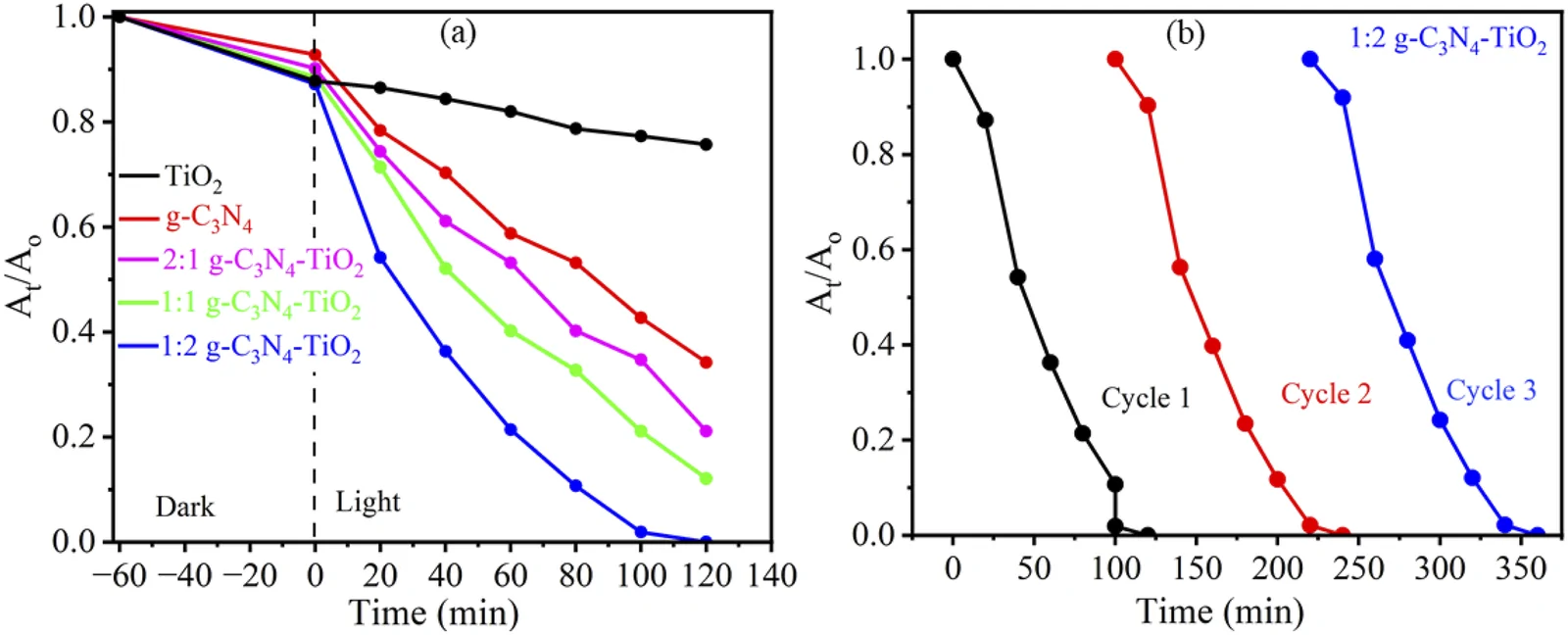
(a) Comparison of catalytic activities of g-C_3_N_4_–TiO_2_ samples. The 1 : 2 g-C_3_N_4_–TiO_2_ exhibited the best degradation efficiency, achieving complete removal of rhodamine B within 120 min due to improved separation of charges. (b) Cycling stability test of the 1 : 2 g-C_3_N_4_–TiO_2_.

The photocatalytic properties of fabricated composites and individual components were investigated based on their ability to remove rhodamine B from aqueous solution under illumination. The degradation efficiency was expressed in terms of *A*_*t*_/*A*_0_ (*A*_0_ and *A*_*t*_ represent the initial absorbance and absorbance at different time intervals, respectively) as a function of time. The observed findings are given in Fig. 7(a). The gradual decrease in *A*_*t*_/*A*_0_ with the passage of time shows the continuous degradation of the rhodamine B dye. About 65, 87, 100, and 78% of the rhodamine B dye (50 ppm, 35 mL) degraded after 2 h reaction with g-C_3_N_4_, 1 : 1 g-C_3_N_4_–TiO_2_, 1 : 2 g-C_3_N_4_–TiO_2_, and 2 : 1 g-C_3_N_4_–TiO_2_, respectively. About 22% decrease in the concentration of dye was observed in the presence of TiO_2_, which is mainly due to adsorption.

**Fig. 8:**
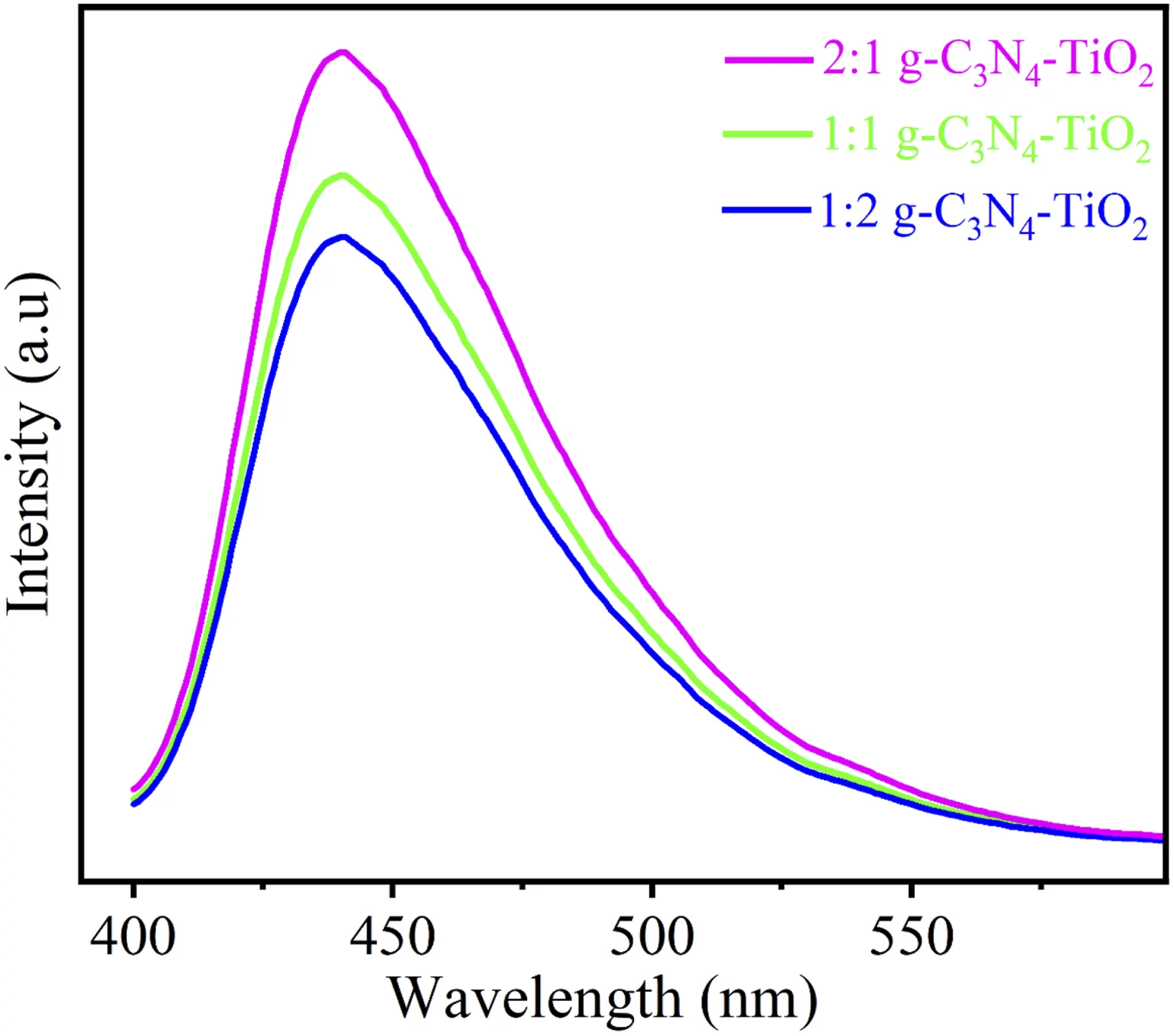
Photoluminescence analysis of g-C_3_N_4_–TiO_2_ samples. Lower PL intensity indicates suppressed recombination of charges. The weak emission intensity with 1 : 2 g-C_3_N_4_–TiO_2_ indicates efficient separation of the charges.

The photocatalytic activity was significantly enhanced through integration of g-C_3_N_4_ with TiO_2_. Such enhancement is associated with the favorable interfacial interactions between the two materials, which promote efficient charge transfer. Within the heterostructure, g-C_3_N_4_ is responsible for visible-light absorption, whereas TiO_2_ receives the excited electrons, thereby enhancing the separation of charges. As a result, the absorption of g-C_3_N_4_–TiO_2_ is broadened to the visible light region, and overall catalytic performance is improved. Among the composites, the 1 : 2 g-C_3_N_4_–TiO_2_ performed best in catalytic activity. The highest performance of the 1 : 2 g-C_3_N_4_–TiO_2_ sample is due to optimized heterojunction formation at this composition and efficient transfer of charges, thereby decreasing the recombination of charges. The separation of the charges was confirmed by photoluminescence analysis (Fig. 8) of g-C_3_N_4_–TiO_2_ samples. The PL emission intensity depends on the recombination rate of charge carriers. The strength of the photoluminescence signals was maximum for the 2 : 1 composite and minimum for the 1 : 2 composite. It shows that the rate of recombination of the charges is highest for the 2 : 1 composite and lowest for the 1 : 2 composite. Hence, the photocatalytic performance is highest for 1 : 2 composite and lowest for the 2 : 1 composite.^35^ Based on the highest activity of 1 : 2 g-C_3_N_4_–TiO_2_, it is suggested that a higher load of TiO_2_ provides more effective electron sinks, resulting in an efficient separation of charges through the flow of electrons towards TiO_2_. The transfer of electrons increases the lifetime of the charges, thereby increasing the generation of reactive species.

**Fig. 9:**
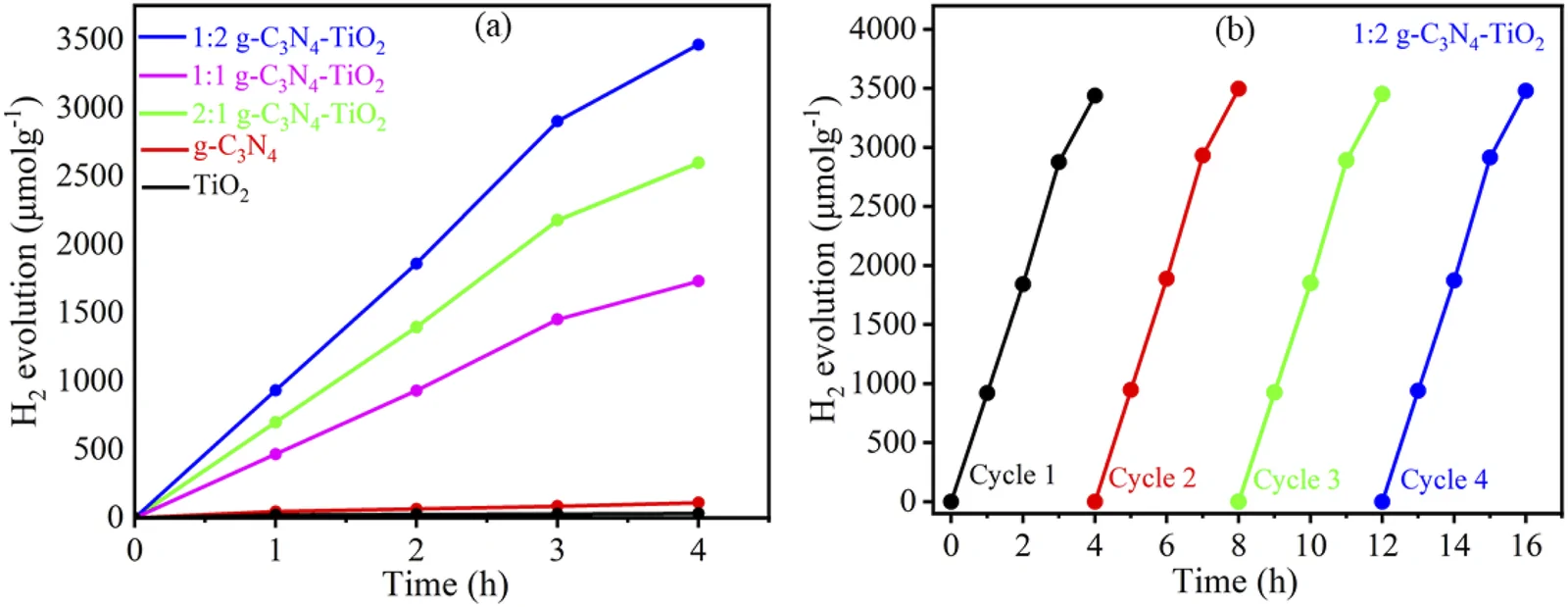
(a) Photocatalytic production of hydrogen. The 1 : 2 g-C_3_N_4_–TiO_2_ heterojunction achieves the highest hydrogen evolution performance. (b) Reusability and stability of the photocatalyst for hydrogen evolution of 1 : 2 g-C_3_N_4_–TiO_2_.

The prepared substances were tested as photocatalysts in hydrogen production as well. The visible light-assisted production of hydrogen from water vapor splitting over g-C_3_N_4_–TiO_2_ samples and individual components is given in Fig. 9(a). The analysis revealed 32, 110, 2597, 3463, and 1791 µmol g^−1^ production of hydrogen with TiO_2_, g-C_3_N_4_, 1 : 1 composite, 1 : 2 composite, and 2 : 1 composite, respectively. The findings revealed that coupling g-C_3_N_4_ with TiO_2_ markedly boosted the photocatalytic efficiency for hydrogen generation.

The photocatalyst showed good stability. It maintained its performance after three dye degradation cycles, Fig. 7(b), and four hydrogen production cycles, Fig. 9(b). These results demonstrate its excellent stability and reusability. The efficient activity of g-C_3_N_4_–TiO_2_ in both reactions, degradation of RhB and hydrogen production, is attributed to enhanced separation of the photoinduced charges. The effectiveness of the present g-C_3_N_4_–TiO_2_ was assessed in relation to analogous systems reported in the literature (Table S2).

### 3.3 Charge transfer properties and photocatalytic mechanism

**Fig. 10:**
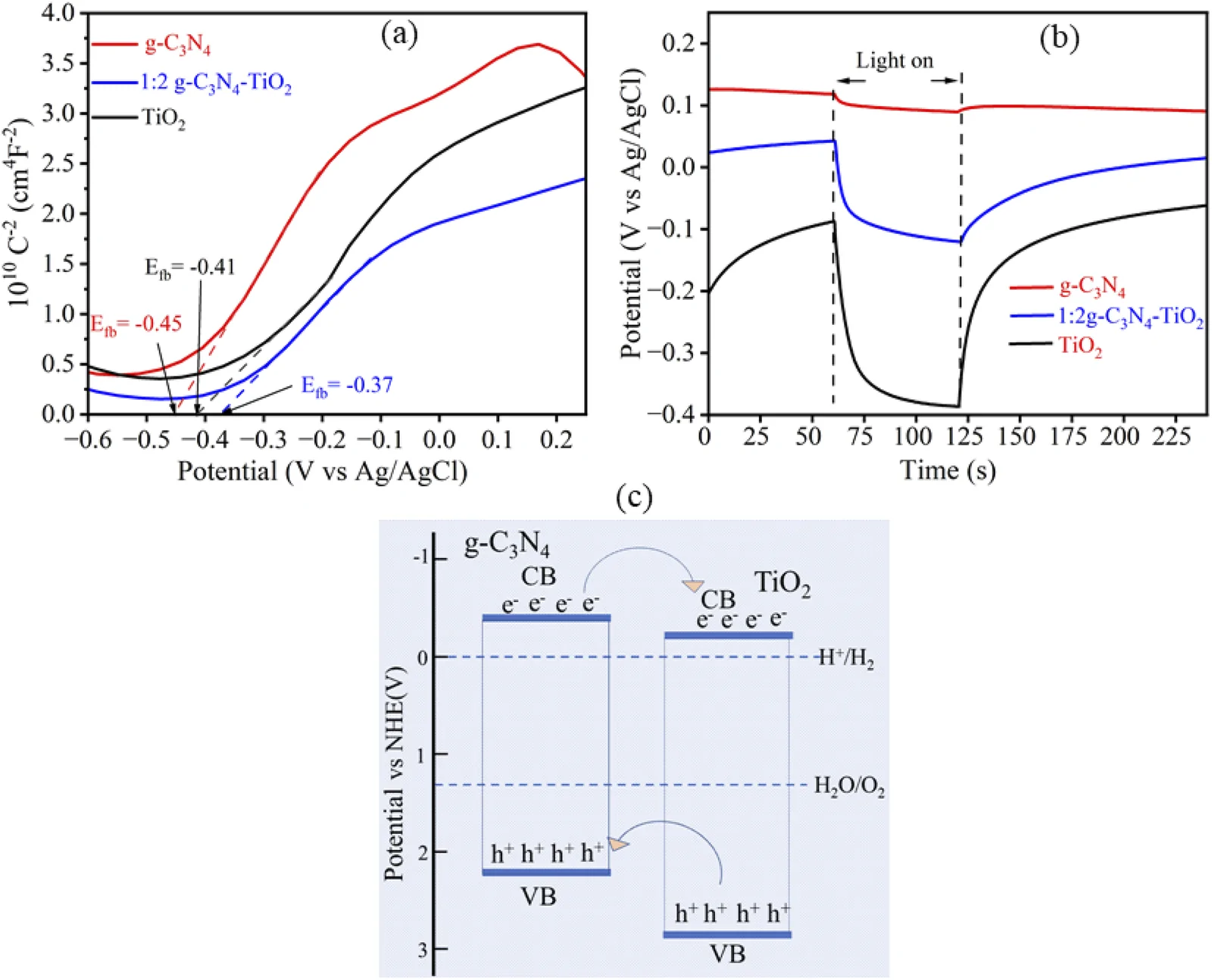
(a) Mott–Schottky plots. (b) Open-circuit potential decay (OCPD) curves. (c) Band alignment diagram derived from electrochemical and optical results.

The Mott–Schottky plots (Fig. 10(a)) exhibited positive slopes, confirming the n-type character of both g-C_3_N_4_ and TiO_2_.^36^ The flat-band potentials (*V*_fb_) were determined as (−0.25 and −0.21 V *vs.* NHE), for g-C_3_N_4_ and TiO_2_, respectively. The more negative Fermi level for g-C_3_N_4_ promotes the migration of electrons towards TiO_2_.^37^ Using the Mott–Schottky analysis and donor density calculations, the positions of conduction band edge for g-C_3_N_4_ and TiO_2_ were estimated as −0.39 and −0.27 V *vs.* NHE, respectively. Based on these values and UV-Vis analysis, the corresponding valence band positions were determined to be 2.3 V and 2.9 V *vs.* NHE (Fig. 4). Additionally, for 1 : 2 g-C_3_N_4_–TiO_2_, *V*_fb_ was positioned at −0.17 V *vs.* NHE. This shift to a more positive potential compared to both g-C_3_N_4_ and TiO_2_ indicates a charge transfer from g-C_3_N_4_ to TiO_2_, supporting the formation of a Type-II heterostructure. Thus, the resulting band alignment is consistent with the formation of a Type-II heterojunction (Fig. 10(c)), in which photogenerated electrons preferentially migrate from CB of g-C_3_N_4_ to that of TiO_2_, while holes are transferred from VB of TiO_2_ to g-C_3_N_4_, promoting efficient spatial separation of charge carriers.^36–38^

The open-circuit potential decay (OCPD) is presented in Fig. 10(b). ΔOCP (OCP_light_ − OCP_dark_) values of 28.7, 297.6, and 162.0 mV were obtained for g-C_3_N_4_, TiO_2_, and 1 : 2 g-C_3_N_4_–TiO_2_, respectively, confirming the distinct charge carrier dynamics of the heterojunction and its ability to promote more efficient charge separation. OCP decay constants (*τ*_2_) were also calculated from eqn (2), once the light was chopped off, resulting in 5.1, 28.2, and 37.0 s for g-C_3_N_4_, TiO_2_, and 1 : 2 g-C_3_N_4_–TiO_2_, respectively. The longer lifetime observed for the heterostructure indicates suppressed electron–hole recombination and enhanced charge carrier retention, which are beneficial for photocatalytic reactions.^36^

## 4 Conclusions

g-C_3_N_4_–TiO_2_ photocatalysts having a composition ratio of 1 : 1, 1 : 2, and 2 : 1 were fabricated using a facile combination of thermal treatment and sol–gel method. The formation of composites was verified with XRD, SEM, UV-DRS, XPS, and FTIR. The prepared heterojunction exhibited improved performance towards degradation of rhodamine B dye and production of hydrogen. The composite with a 1 : 2 ratio of g-C_3_N_4_ and TiO_2_ exhibited superior performance among all. Almost completely rhodamine B dye (50 ppm, 35 mL) degraded after 2 h reaction with 1 : 2 g-C_3_N_4_–TiO_2_. Similarly, 3463 µmol g^−1^ hydrogen was produced using 1 : 2 g-C_3_N_4_–TiO_2_ as a catalyst. The study demonstrates that constructing a g-C_3_N_4_–TiO_2_ Type II heterojunction significantly improves catalytic activity due to improved carrier dynamics, making it a promising approach for sustainable wastewater treatment and solar-driven hydrogen production.

## Author contributions

Conceptualization: Marcus Vinicius Castegnaro; Muhammad Saeed, Sherdil Khan; methodology: Asif Jamil; Sherdil Khan; formal analysis and investigation: Asif Jamil; Muhammad Rameez Khan Khattak; Anderson Thesing; Larissa Stein Godoi; Giovanna Machado; writing – original draft preparation: Asif Jamil; Muhammad Saeed; writing – review and editing: Asif Jamil; Marcus Vinicius Castegnaro; Muhammad Saeed; Larissa Stein Godoi; Sherdil Khan.

## Conflicts of interest

The authors have no financial or proprietary interest in any material discussed in this article.

## Supplementary Material

RA-016-D6RA03962F-s001

## Data Availability

All the data obtained during experimentation is given in the manuscript. Supplementary information (SI): (1) optical absorption spectra of TiO_2_, g-C_3_N_4_, and g-C_3_N_4_–TiO_2_ composites, (2) XPS survey spectra of 1 : 1 g-C_3_N_4_–TiO_2_, 1 : 2 g-C_3_N_4_–TiO_2_, and 2 : 1 g-C_3_N_4_–TiO_2_, composites, (3) Table S1. Parameters obtained from the biexponential fitting of the open-circuit potential decay (OCPD) curves, (4) Table S2. Photocatalytic activities of various catalysts reported in literature. See DOI: https://doi.org/10.1039/d6ra03962f.
