# Supplementary material for: Development of g-C3N4–TiO2 heterojunction for photocatalytic degradation of rhodamine B dye and production of hydrogen
Source: RSC Adv. 2026 Aug 28;16(42):50942–50. doi: 10.1039/d6ra03962f (PMC13522948; doi:10.1039/d6ra03962f)
Supplement: Supplementary file 1 [file RA-016-D6RA03962F-s001.pdf]

Supplementary Information (SI) for RSC Advances.
This journal is © The Royal Society of Chemistry 2026

Development of g-C_3N_4-TiO_2 heterojunction for photocatalytic degradation of Rhodamine B dye and production of hydrogen

Asif Jamil[1], Marcus Vinicius Castegnaro[1*], Muhammad Saeed[2], Muhammad Rameez Khan Khattak[1], Anderson Thesing[1], Larissa Stein Godoi, Giovanna Machado[3], Sherdil Khan[1*]

[1]Institute of Physics, Universidade Federal do Rio Grande do Sul, Av. Bento Gonçalves 9500, Bairro Agronomia, Porto Alegre, RS 91501-970, Brazil

[2]Department of Chemistry, Government College University Faisalabad, Faisalabad, Pakistan

[3]Laboratory of Microscope and Microanalysis, Northeast Center for Strategic Technologies (CENETE), Avenida Prof. Luís Freire, 1, Cidade Universitária, Recife, Pernambuco 50740-540, Brazil

*Corresponding author: marcus.castegnaro@ufrgs.br, sherdil.khan@ufrgs.br

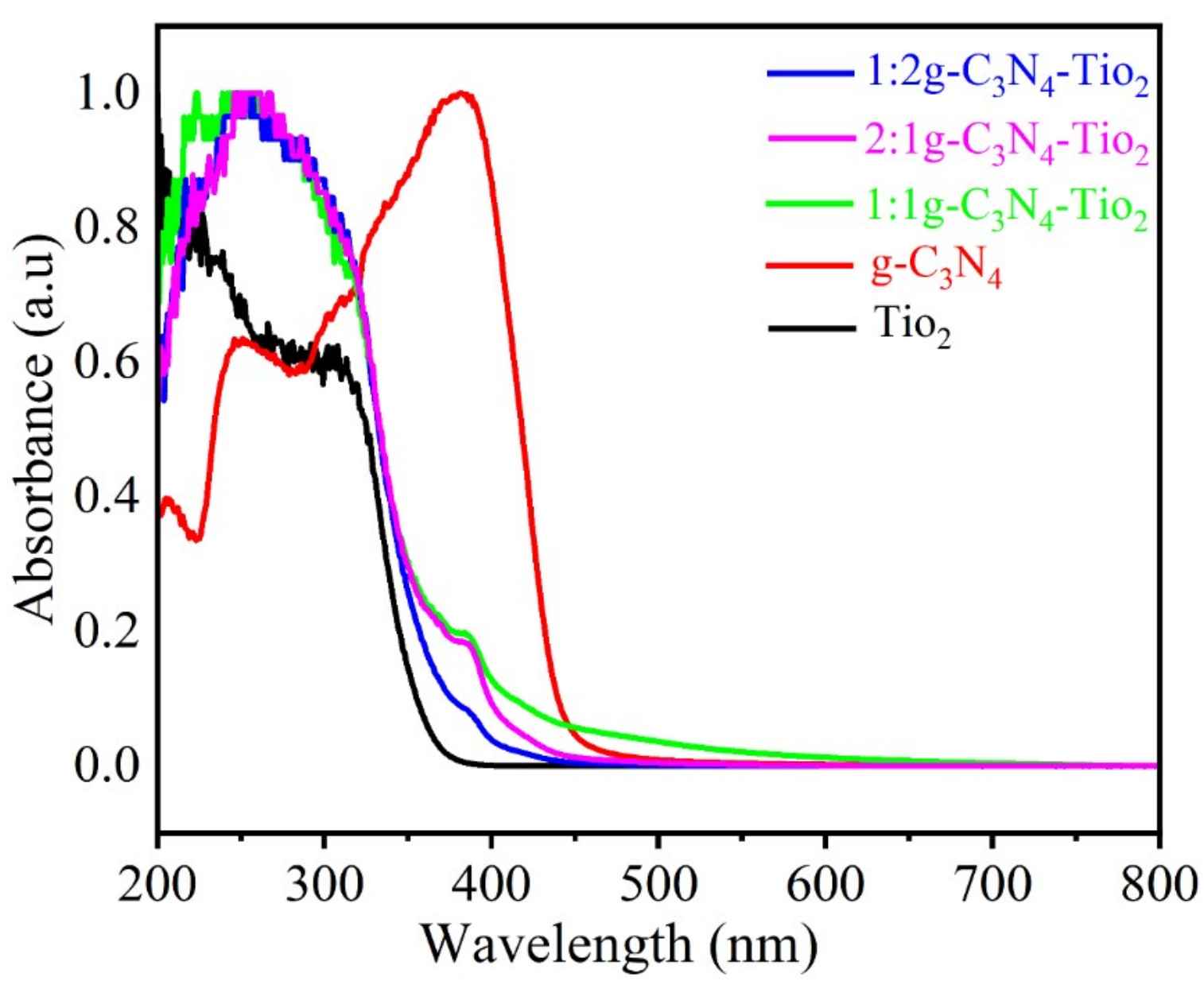

Figure S1: Optical absorption spectra of TiO_2, g-C_3N_4, and g-C_3N_4 -TiO_2 composites with different g-C_3N_4 -TiO_2 mass ratios (1:1, 2:1,1:2).

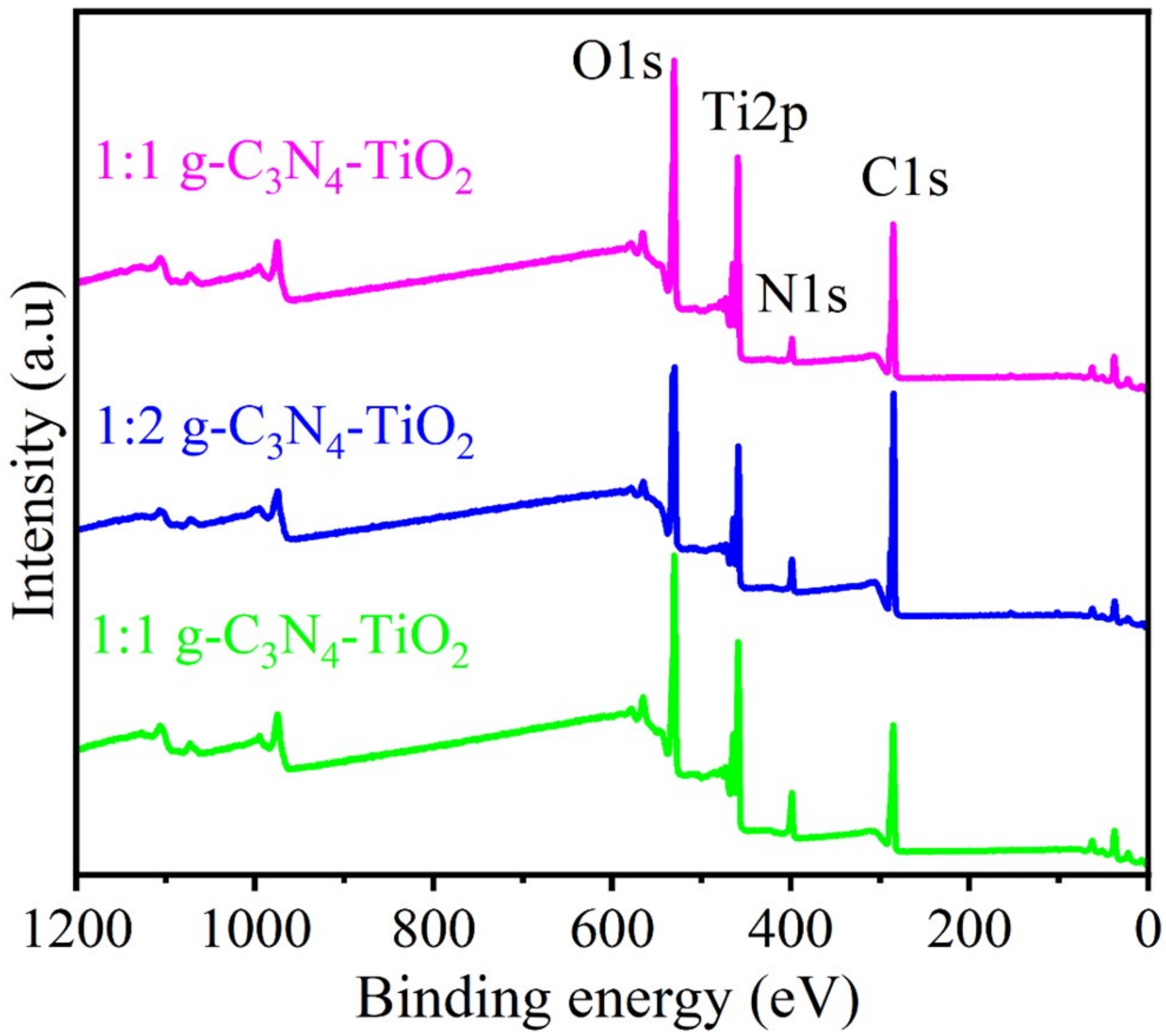

Figure S2 XPS survey spectra of 1:1 g-C_3N_4-TiO_2, 1:2 g-C_3N_4-TiO_2, and 2:1 g-C_3N_4-TiO_2, composites.

Table S1. Parameters obtained from the biexponential fitting of the open-circuit potential decay (OCPD) curves, including the characteristic decay constants related to recombination of the photogenerated charge carriers and their corresponding uncertainties.

**Samples**	τ_1**(s)**	τ_2**(s)**
g-C_3N_4	1.4±0.12	5.1±0.15
TiO_2	3.8±0.04	28.2±0.42
1:2 g-C_3N_4-TiO_2	2.4± 0.03	37.0±0.10

Table S2 Photocatalytic activities of various catalysts reported in literature

S. No	Substrate	Catalyst	Activity	Reference
1	Rhodamine B dye (50 ppm, 35 mL); 120 min reaction	$g\text{-}C_3N_4\text{-}TiO_2$	100%	This study
2	10 mg/L of Rhodamine B, Methylene blue; 60 min reaction	$TiO_2/g\text{-}C_3N_4$	99%	Liu et al.[1]
3	5 mg/L of Rhodamine B; 60 min reaction	$g\text{-}C_3N_4/TiO_2$	97%	Monga & Basu[2]
4	5 mg/L of Methyl orange; 120 min reaction	$g\text{-}C_3N_4$	>95%	Xu et al.[3]
5	5 mg/L of Methyl orange, methylene blue, Rhodamine B; 75 min reaction	$g\text{-}C_3N_4$	>95%	Nabi et al.[4]
6	10 mg/L of Rhodamine B; 120 min reaction	$TiO_2/g\text{-}C_3N_4$	90%	Premalatha et al.[5]
7	20 mg/L of Methyl orange; 80 min reaction	$TiO_2/g\text{-}C_3N_4$	98%	Huang et al.[6]
8	12 µM of Rhodamine B; 20 min reaction	$g\text{-}C_3N_4\text{- }TiO_2$	90%	Sharma et al.[7]
9	50 mg/L of Tetracycline; 300 min reaction	PVDF-TiO_2@g-C_3N_4	97%	Zheng et al.[8]
10	20 mg/L of Brilliant green, 150 min reaction	$g\text{-}C_3N_4\text{- }TiO_2$	100%	Sutar et al.[9]
11	10 mg/L of Phenol; 60 min reaction	$TiO_2/g\text{-}C_3N_4$	>90%	Wei et al.[10]
12	10 mg/L of Methylene blue; 180 min reaction	$g\text{-}C_3N_4/TiO_2$	100%	Gundogmus et al.[11]
13	25 mg/L of Methylene blue; 80 min reaction	$TiO_2/g\text{-}C_3N_4$	73%	Girish et al.[12]
14	10 mg/L of Methylene blue; 180 min reaction	$g\text{-}C_3N_4/TiO_2$	70%	Boonprakob et al.[13]
15	Triethanolamine solution	$g\text{-}C_3N_4\text{-}TiO_2$	3463 µmol g^{-1} of H_2	This study

16	Glucose reforming for hydrogen production	$g\text{-}C_3N_4/TiO_{2-X}$	1104.15 $\mu mol\cdot g^{-1}\cdot h^{-1}$ of H_2	Wang et al.[14]
17	Ethanol solution	$TiO_2/g\text{-}C_3N_4$	16.2 mmol g^{-1} h^{-1} of H_2	Mtunzi et al.[15]
18	Lactic acid	TiO_2–$g\text{-}C_3N_4$–rGO	27.40 mmol$g^{-1}h^{-1}$ of H_2	Raja et al.[16]
19	Methanol solution	$TiO_2/g\text{-}C_3N_4$	5.8 $mmol\cdot g^{-1}\cdot h^{-1}$ of H_2	Li et al.[17]
20	Methanol solution	$Cu\text{-}TiO_2/g\text{-}C_3N_4$	10.21 mmol g^{-1} h^{-1} of H_2	Wang et al.[18]

References

1 J. Liu, J. Zheng, G. Yue, H. Li, Z. Liu, Y. Zhao, N. Wang, C. Sun and Z. Cui, *RSC Adv.*, 2022, **12**, 10258–10266.

2 D. Monga and S. Basu, *Advanced Powder Technology*, 2019, **30**, 1089–1098.

3 H. Y. Xu, L. C. Wu, H. Zhao, L. G. Jin and S. Y. Qi, *PLoS One*, DOI:10.1371/journal.pone.0142616.

4 G. Nabi, N. Malik, M. B. Tahir, M. Tanveer, R. Rafique, S. Rehman, R. Yousaf and S. Akram, *Int. J. Environ. Anal. Chem.*, DOI:10.1080/03067319.2020.1849666.

5 N. Premalatha, P. Rajalakshmi and L. R. Miranda, *Reaction Kinetics, Mechanisms and Catalysis*, DOI:10.1007/s11144-022-02154-5.

6 Z. Huang, S. Jia, J. Wei and Z. Shao, *RSC Adv.*, 2021, **11**, 16747–16754.

7 M. Sharma, S. Vaidya and A. K. Ganguli, *J. Photochem. Photobiol. A Chem.*, 2017, **335**, 287–293.

8 X. Zheng, Y. Liu, X. Liu, Q. Li and Y. Zheng, *Ecotoxicol. Environ. Saf.*, DOI:10.1016/j.ecoenv.2020.111866.

9 R. S. Sutar, R. P. Barkul, S. D. Delekar and M. K. Patil, *Arabian Journal of Chemistry*, 2020, **13**, 4966–4977.

10 H. Wei, W. A. McMaster, J. Z. Y. Tan, L. Cao, D. Chen and R. A. Caruso, *Journal of Physical Chemistry C*, DOI:10.1021/acs.jpcc.7b06493.

11 P. Gündoğmuş, J. Park and A. Öztürk, *Ceram. Int.*, DOI:10.1016/j.ceramint.2020.05.241.

12 Y. R. Girish, Udayabhanu, G. Alnaggar, A. Hezam, M. B. Nayan, G. Nagaraju and K. Byrappa, *Journal of Science: Advanced Materials and Devices*, DOI:10.1016/j.jsamd.2022.100419.

13 N. Boonprakob, N. Wetchakun, S. Phanichphant, D. Waxler, P. Sherrell, A. Nattestad, J. Chen and B. Inceesungvorn, *J. Colloid Interface Sci.*, 2014, **417**, 402–409.

14 Y. Wang, R. Shi, Z. Wei, H. H. Liu, L. Xie, G. Du and X. Chai, *Fuel*, 2027, **427**, 139647.

15 F. M. Mtunzi, N. D. Shooto, B. J. Okoli, J. Kumar and T. G. Gindose, *J. Photochem. Photobiol.*, 2026, **31**, 100273.

16 A. Raja, R. K. Chava and M. Kang, *Chemical Engineering Journal*, 2025, **513**, 163000.

17 R. Li, J. Peng, X. Yu, Q. Xi, J. Qin, J. Liu and J. Wang, *Inorg. Chem. Commun.*, 2025, **182**, 115665.

18 C. Wang, Y. Sun, Z. Wang, Y. Zeng, Q.-Y. Li, X. Kang and G. Huang, *Green Energy & Environment*, DOI:10.1016/j.gee.2026.01.008.
